# The Priest, the Sex Worker, and the CEO: Measuring Motivation by Job Type

**DOI:** 10.3389/fpsyg.2020.01321

**Published:** 2020-06-19

**Authors:** Jan Ketil Arnulf, Kim Nimon, Kai Rune Larsen, Christiane V. Hovland, Merethe Arnesen

**Affiliations:** ^1^Department of Leadership and Organizational Behaviour, BI Norwegian Business School, Oslo, Norway; ^2^Department of Human Resource Development, The University of Texas at Tyler, Tyler, TX, United States; ^3^Leeds Business School, University of Colorado at Boulder, Boulder, CO, United States

**Keywords:** motivation, semantic theory of survey response, Likert scale analysis, job types, job design theory, self-determination theory, latent semantic analysis

## Abstract

This study uses latent semantic analysis (LSA) to explore how prevalent measures of motivation are interpreted across very diverse job types. Building on the Semantic Theory of Survey Response (STSR), we calculate “semantic compliance” as the degree to which an individual’s responses follow a semantically predictable pattern. This allows us to examine how context, in the form of job type, influences respondent interpretations of items. In total, 399 respondents from 18 widely different job types (from CEOs through lawyers, priests and artists to sex workers and professional soldiers) self-rated their work motivation on eight commonly applied scales from research on motivation. A second sample served as an external evaluation panel (*n* = 30) and rated the 18 job types across eight job characteristics. Independent measures of the job types’ salary levels were obtained from national statistics. The findings indicate that while job type predicts motivational score levels significantly, semantic compliance as moderated by job type job also predicts motivational score levels usually at a lesser but significant magnitude. Combined, semantic compliance and job type explained up to 41% of the differences in motional score levels. The variation in semantic compliance was also significantly related to job characteristics as rated by an external panel, and to national income levels. Our findings indicate that people in different contexts interpret items differently to a degree that substantially affects their score levels. We discuss how future measurements of motivation may improve by taking semantic compliance and the STSR perspective into consideration.

## Introduction

“Most social acts have to be understood in their setting, and lose meaning if isolated. No error in thinking about social facts is more serious than the failure to see their place and function” Asch (1987).

Asch’s (1987, p. 61, orig. 1952) warning is as relevant today as half a century ago. The numbers emerging from Likert-scale data are what social anthropologist Geertz (1973) called “thin data” because they reduce a complex experience to seemingly uniform rows of numbers. The meaning of these numbers is still debated in the methodological literature (Drasgow et al., 2015; Kjell et al., 2019). From a linguistic point of view, it is unlikely that short sentences of the type normally used in Likert-scale items will mean the same to all people regardless of the context of the respondents (Kay, 1996; Borsboom, 2008; Maul, 2017). To the extent that people interpret items differently according to their own situations, the item texts function like a story, where the items combine in different ways to describe different contexts.

This study explores how people responding to the same items about motivation seem to interpret these in different ways dependent on their professional contexts, a phenomenon not accounted for in most theories, and not part of standard psychometrics. Our aim is to show how item interpretation may be almost be as deep a characteristic of different groups as the score levels themselves.

Recent developments in quantitative text analysis suggest that quantitative responses to survey items may be heavily influenced by semantics (e.g., Nimon et al., 2016; Rosenbusch et al., 2019; Arnulf and Larsen, 2020). The Semantic Theory of Survey Response (STSR) claims that the most obvious reason for covariation between items is that they are semantically related (Arnulf et al., 2018d). Empirical testing of STSR has revealed that the correlation matrix of survey data can be strongly determined by their semantic properties, but not always, and not necessarily to the same extent across all groups (e.g., Arnulf et al., 2014; Nimon et al., 2016). In the present study, we examine whether interpretation of the same item sets differs systematically across contextually consistent respondent sets. For example, will items on the motivational effects of payments mean the same regardless of the expected income of people?

A study by Drasgow et al. (2015) expressed doubts about interpreting Likert-scale measurements as “dominant measures” where all traits are uniformly scalable from low to high. Instead, they suggested that respondents display preferred values, choosing alternatives that more accurately describe their viewpoints but not necessarily on a more-or-less continuum. A similar argument has been raised in a study that used semantic algorithms to rate free-text responses in a personality survey (Kjell et al., 2019).

The purpose of this study is therefore to explore the degree to which subjects from different professional contexts respond to motivational items in ways that cohere with or deviate from what is semantically expected. The contributions of this study are to: (a) strengthen STSR by establishing a technique for assessing the mutual impact of score levels and semantic characteristics of items in differentiating between groups of respondents, (b) contribute a general understanding of the psychology involved in item responses for different occupational groups, and (c) advance ways to use semantic algorithms as a methodological tool in social sciences including and not limited to organizational behavior and social psychology.

## Theory

In his original description of the scales that now carry his name, Likert (1932, p. 7, italics in orig.) wrote: “…it is strictly true that the number of attitudes which any given person possesses is almost infinite. This result is statistically as well as psychologically absurd. Exactly the same absurdity and the same obstacle to research is offered by those definitions of attitude which conceive them merely as *verbal expressions*…”

Now, almost 100 years later, working with the verbal expressions is no longer an absurdity, neither statistically nor psychologically. Language algorithms have opened a way to work precisely with the self-descriptive statements that Likert (1932) and his contemporaries could not address (Nimon et al., 2016; Arnulf et al., 2018a; Kjell et al., 2019). Our basic assumption in this study builds on the linguistic fact that all worded statements mean different things to different subjects dependent on their context (Kay, 1996; Sidnell and Enfield, 2012). An example of this has previously been described by Putnick and Bornstein (2016) who noted that symptoms of depression such as crying are different between men and women, influencing the score levels on questions with such content. Similarly, our focus here is on how questions about motivation may take on different meanings in different job types, affecting score levels.

In what follows, we suggest that the common approach to treating survey responses as measures builds on an incomplete understanding of the meaning of the numbers with respect to their semantic dependencies. We then argue that semantic analysis is a viable approach to a different appreciation of survey items that may possibly alleviate some of the previously described problems. The arguments will be tested in an empirical analysis of a dataset containing self-rated motivation across very different professional contexts to support our claims. To finally ascertain that the semantic influence is not methodological artifact, we will validate the semantic data with two other independent data sources, an independent rating panel and national income statistics.

### Likert Scale Measures of Contextual Motivation

Likert’s (1932) argument for trusting the numbers from his scales was that working with verbal expressions would be methodologically impossible. Hence, the simplification of attitudes imposed on responses by using numerical scales was the only workable solution for empirical research. To this day, history has judged Likert right and the use of his scales is one of the most commonly used measurement instruments in social science research and enables a range of practical applications (Likert, 1932; Podsakoff et al., 2003; Sirota et al., 2005; Cascio, 2012; Yukl, 2012a; Lamiell, 2013).

Despite or perhaps even because of its intuitive simplicity, however, other researchers have been critical of some of the uses of Likert scales since the days of its conception (e.g., Andrich, 1996; Drasgow et al., 2015). One problematic aspect of Likert scales is that while the response categories are usually framed as texts, they are transformed into numbers used for calculations. These numbers are in turn translated back to texts as inferences about the measured attitudes (Kjell et al., 2019). An item with the text “I will look for a new job in the next year” may be scored as “Definitely not – probably not – maybe – probably – definitely yes.” The choice of the option “I will definitely look for a new job in the next year” would be assigned a numerical value (e.g., 5) as a measure used for calculations. Following a commonly used convention, “measurement” can be defined as the “process of assigning numbers to represent qualities” (Campbell, 1920, p. 267). However, it is not entirely obvious what numbers from Likert scales measure (Smedslund, 1988; Elster, 2011; Mari et al., 2017; Slaney, 2017). While measurement is a complex concept that can be defined in numerous ways, some conventionality in the definition of measurement seems unavoidable (Mari et al., 2017, pp. 117–121). The conventionality or common sense element seems to require that a “measure” should retain its meaning across contexts in order to be a valid measurement. We expect measurement units of walls and floors to be consistent independently of the size of buildings and expect temperature assessments to allow comparisons of polar with tropical environments. Such invariance does not necessarily apply to numbers from Likert scale items. “A warm day” refers to very different measured temperatures in Texas and Norway, and used as a survey item, the distinction between contexts may blur. The same problem could possibly arise with measurements in social science. Will the same statement about motivation imply the same attitudinal measure across contexts? Or will “satisfaction with pay” mean different things dependent on the difference in payment levels between job types?

The STSR offers a framework to test these questions empirically. The theory posits that for two different items to be scored independently, they also need to be semantically independent. If two items are semantically intertwined, the answer to the second will somehow depend on the first – unless the respondents make different interpretations of the items (Schwarz, 1999). It is this difference that we can try to assess using the semantic techniques that we will explain below.

*Motivation* is a latent variable (Borsboom, 2008). Assessments of *motivational strength* are therefore not directly accessible, even to the individual in question (McClelland et al., 1989; Parks-Stamm et al., 2010). Thus, self-rated motivation is likely to be influenced by a number of factors. However, a large number of studies on motivation in the workplace have relied on Likert-scale items to model motivational effects. Among these studies, two theories stand out as particularly relevant to our aim: The Job Characteristics Model (JCM) originally proposed by Hackman and Oldham (1976) proposed that different job contexts – their characteristics – would have systematically different impacts on employee motivation. A later development, the Self-Determination Theory (SDT) built on this and outlined how contextual variables could translate into types of motivation that enhance or impair performance (Deci et al., 1989, 2017). Building on these traditions, Barrick et al. (2013) outlined how individual characteristics interact with situational variables in a sense-making process to create different types of job motivation through experiencing work as meaningful. We can thus build a framework of theory and existing research to assess the impact of semantics on survey responses in work motivation:

Our first interest concerns work contexts as we assume that these will impact motivational levels *as well as* the interpretations of items. The Job Characteristics Model (JCM) (Hackman and Oldham, 1975, 1976) has been in prevalent use for work design over two decades (Kanfer et al., 2017, p. 342). Precisely because JCM focuses on *job characteristics*, the model should help us identify aspects of jobs that are inter-subjectively valid and not indicative of individual differences between employees. In fact, the origins of JCM was an explicit intention to identify situational variables such that one may measure the impact of job design on motivation (Hackman and Oldham, 1976, p. 252).

According to JCM, five *core* job dimensions will affect motivation: (a) skill variety, (b) task identity, (c) task significance, (d) autonomy, and (e) feedback (Hackman and Oldham, 1975, 1976). We therefore assume that these dimensions will be important descriptors of jobs where subjects may vary in types and levels of motivation as well as in their interpretation of items. Expanding on these, later research has identified enriched social roles, influence and status as belonging to taxonomies of job situations (Oldham and Hackman, 2010; Barrick et al., 2013).

The JCM theory presumes that job characteristics will interact with different needs in the different employees to induce levels of motivation (Hackman and Oldham, 1976). This subjective interpretive process has been elaborated in more detail by Self-Determination Theory (SDT) (Deci et al., 1989, 2017; Ryan and Deci, 2000a, b), and has served as framework for research on motivation and work outcomes using self-perception with Likert-type rating scales (Grant, 2008; Dysvik et al., 2010; Fang and Gerhart, 2012; Rockmann and Ballinger, 2017).

According to SDT, conditions that activate motivation can be distinguished on a continuum from autonomous to controlled, where controlled types of motivation are less favorable: “external regulation can powerfully motivate specific behaviors, but it often comes with collateral damage in the form of long-term decrements in autonomous motivation and well-being, sometimes with organizational spillover effects” (Deci et al., 2017, p. 21). Instead, autonomous motivation – where intrinsic motivation (IM) or pleasure in the activity for its own sake is one type, tends to have better outcomes: “Employees can be intrinsically motivated for at least parts of their jobs, if not for all aspects of them, and when intrinsically motivated the individuals tend to display high-quality performance and wellness” (Deci et al., 2017, p. 21).

As can be seen from the explanations above, SDT does not assume an automatic relationship between situational context and type of motivation. Rather, the sub-optimal effect of extrinsic motivation is linked to a perception of being controlled. Also, IM is not always assumed to induce superior performance to extrinsic motivation. Still, the aim of the theory is to guide managerial practices that facilitate intrinsic types of motivation, because these are generally seen to produce better outcomes. The relationship to situations is clearly outlined in a recent summary of research in the field (Deci et al., 2017, p. 20): “Some have careers that are relatively interesting and valued by others. Their work conditions are supportive, and they perceive their pay to be equitable. Others, however, have jobs that are demanding and demeaning. Their work conditions are uncomfortable, and their pay is not adequate for supporting a family. They are likely to look forward to days away from work to feel alive and well.” The cited summary reviews a number of studies that show how extrinsic rewards may reduce performance through experience of being controlled, and how IM generally leads to better performance in terms of effort, quality, and subjective wellness.

The final point to be elaborated is the interpretive process that translates the job characteristics into the experienced motivational states. Outlining a “theory of purposeful behavior,” Barrick et al. (2013, p. 149) claimed that individuals take an agentic, proactive role in “striving for …higher-order goals and experienced meaningfulness associated with goal fulfillment.” They argued that individual characteristics and higher-order goals interact to make performance at work meaningful. The authors cite the work of Weick on sensemaking (e.g., Weick, 1995, 2012), who explained how experiences at work are transformed into communicative practice as recursive social interaction. According to Barrick et al. (2013), “employees actively engage in an interpretive process to make meaning of their own jobs, roles, and selves at work by comprehending, understanding, and extrapolating cues received from others” (p. 147).

In other words, the subjectively experienced motivational state is a product, first, of the situation, but secondly, of how this situation is interpreted through social sense-making through language. This process should in turn affect the experienced levels of effort and quality exerted at work, together with a general sense of wellness, as experienced in the intention to stay in this job and as commitment to the organization. The chosen framework gives us the opportunity to operationalize situations using JCM and later extensions, predict ratings of motivations and outcomes building on SDT, and explore whether item responses reflect job characteristics, interpretive processes, or both. We want to emphasize here that our main concern is not with the theories of motivation itself, but with the contextually determined interpretation of Likert-scale items. The present theories are chosen for the way they allow exploration of contextual variables that influence text interpretation as well as motivational effects, hence the inclusion of self-rated levels of motivational outcomes.

Since job characteristics and types of motivation have been object of extensive research as quoted above, our focus is on the prospect of exploring the interpretive, semantic process involved to which we now turn.

### Semantic Analysis

Work on natural language parsing in digital technologies has yielded a number of different techniques used with increasing frequency in social science. We will not review these in depth here, but concentrate on a brief description of latent semantic analysis, the technique used in the present study.

Latent semantic analysis (LSA) is a mathematical approach to assessing meaning in language, similar to how the brain determines meaning in words and expressions (Landauer and Dumais, 1997; Kintsch, 2001; Dennis et al., 2013). The general principle behind LSA is that the meaning of any given word (or series of words) is given by the contexts where this word is usually found. Just as children pick up the meaning of terms by noticing how they are applicable across different situations, LSA is a mathematical technique for determining the degree to which two expressions are interchangeable in a language.

Latent semantic analysis does this by establishing a semantic space from existing documents such as newspaper stories, journal articles and book fragments. In these semantic spaces, documents are used as *contexts* and the number of times any word appears in each context is entered in a word-by-document matrix. This matrix can be created out of a smaller number of texts, but the best results are typically obtained with semantic spaces containing millions of words in thousands of documents (Dumais et al., 1988; Landauer and Dumais, 1997; Gefen et al., 2017). From here, LSA transforms the sparse word-by-document matrix into three new matrices through singular value decomposition, a technique similar to principal component analysis (Günther et al., 2015; Gefen et al., 2017). Finally, researchers may project new texts of interest into these matrices to obtain a numerical estimate for the degree to which they are similar in meaning.

In a series of recent studies, LSA techniques have been used to explore a range of phenomena in survey statistics. Correlations between constructs have been explained as a result of semantic overlap (Nimon et al., 2016), as are the relationships between leadership behaviors and outcomes (Arnulf et al., 2014) and variable relationships in the technology acceptance model (Gefen and Larsen, 2017). In the same way, construct overlap (the so-called “jingle-jangle fallacy”) was demonstrated and possibly empirically validated with the use of LSA (Larsen and Bong, 2016). The technique has also been applied to individual characteristics in responses, such as diagnosing psychopathology (Elvevag et al., 2017; Bååth et al., 2019), establishing personality patterns (Kjell et al., 2019), or predicting individual survey responses (Arnulf et al., 2018b).

One application that we will use here builds on a previous study of how semantically driven respondents are (Arnulf et al., 2018d). The argument in this approach is that strong semantic relationships between items will create higher correlations. An item with the wording “I like my job” will correlate highly with “I enjoy my work” simply because they share the same meaning and the LSA cosine for the two sentences are 0.73. Conversely, for two items to validly obtain different scores, they need to have dissimilar meanings. The LSA cosine for the items “I like my job” and “Customers are demanding” is −0.03, and they are not necessarily correlated even if they sometimes could be.

It is possible then to assess how similar any individual’s set of scores is by calculating the distances between each pair of item scores. This approach has been investigated in four independent samples and was found to correspond to the response pattern predicted by LSA values (Arnulf et al., 2018d). Not all Likert scale instruments are equally semantically determined, and some seem entirely devoid of semantic predictability – the text algorithms may detect patterns but these do not seem to predict patterns in human responses (Arnulf et al., 2014). To the extent that a survey has a demonstrable semantic structure, we can assess the degree to which each single respondent is *compliant* with the semantic structure of the survey. To the degree that people are semantically compliant, they contribute to a response pattern that is semantically predictable, either as individuals or groups.

To compute semantic compliance, we first create a score distance matrix for each individual. The score distance matrix is similar to the correlation matrix for the sample, but consists of the absolute difference in score level between two of the individual’s scores [abs(score1-score2), abs(score1-score3)…]. We can then regress the individual’s score distances on the semantically calculated matrix from LSA (Benichov et al., 2012; cf. Arnulf et al., 2018d). Take the three items used as example above: assume that to the items “I like my job,” “I enjoy my work,” and “Customers are demanding,” our respondent answers 5, 5, and 2. The distance matrix between the three responses would be (5–5 = 0), (5–2 = 3), and (5–2 = 3). The series of LSA cosines 0.73, −0.03, and −0.03 are correlated −1.0 with the score differences (note the negative sign – *higher* overlap in meaning will result in *smaller* score distances).

As an operationalized measure of semantic compliance, we keep the unstandardized slope from the regression for each individual. If we regress the score distances above on the cosines, we get a slope of −3.95. The further from the semantically expected pattern (the weaker the slope), the more the individual may have made a personal interpretation of an item that departs from the semantically expected. We use this unstandardized slope as a measure and operationalization of how closely the single respondent matches a response pattern as predicted by the semantic algorithm alone.

### Hypotheses About the Meaning of Motivational Items

Our unique approach to the measurement of motivation is now based on the combination of two approaches: examination of score levels and semantic compliance across a group of professions with different job characteristics. According to JCM, holders of jobs should display different motivational levels if the characteristics of the job also vary along the dimensions proposed by the theory. In other words, we are looking for response characteristics due to job types instead of individual differences (Hackman and Oldham, 1976; Chiu and Chen, 2005). However we are looking for two types of differences emanating from different job characteristics: The first would be the expected differences in motivational score levels, based on the influence that job characteristics are theoretically supposed have. The second is if different job characteristics will also influence the understanding of survey items in a way that is detectable by text algorithms.

This second type of differences goes back to Likert’s (1932) original claim that verbal statements are beyond methodological reach. If we can begin to explore how different groups of respondents are systematically different in their response patterns, we can expand our tools of measurement beyond the simplification inherent in pure scale values. We can then begin to assess the impact of semantic factors such as context dependence, communities of practice, and social desirability, to name a few. By seeking a wide variation in possible job characteristics, we aimed to explore how semantics would explain the similarities and differences in frequently used measures of subjectively perceived motivation. Our exploration was guided by four hypotheses.

The first possibility we want to explore is if it is possible to show that reported levels of motivation are dependent on how the respondents interpret the items. If this is true, then the motivational levels will not only depend on the job type. The reported level of motivation will also depend on semantic compliance (i.e., differences in interpretation of items). Moreover, since different contexts will influence what the items mean to the respondents, these sources of variance will interact with each other. So, the main purpose of our study can be summed up in as follows:

H1: Self-reported levels of motivation differ by job type and the interaction between job type and semantic compliance.

However, the effects we look for in H1 are all taking place in the same responses – job holders who rate their levels of motivation are also displaying semantic characteristics. This risks a same-source bias, begging the question of which effect might be an artifact of the other (Podsakoff et al., 2012). We therefore want to follow the dynamics of semantics by tracing the effects of semantics to data sources independent of the subjective raters themselves. We start unpacking the problem by a series of hypotheses that relate to independent data. Our first independent data point is the salary level of each profession, not as self-rated but as the levels estimated by the national bureau of statistics in Norway (SSB). There are several reasons for choosing this type of data.

First, the salary levels of a profession in society is linked to the market value of this profession (Obermann and Velte, 2018). The mutual differences between salary levels of professions will be mixed a function of social status and macro-economic evaluation in the job markets, with possible effects on the interpretation of survey items. Secondly, research on JCM and on SDT (Kuvaas, 2006b; Deci et al., 2017) shows that monetary rewards have complicated effects on motivation its outcomes on work. Payment systems may exert a negative effect through perceptions of external control and counter-productive work focus. On the other hand, higher level of payment may signal recognition, status and power in ways that were predicted to increase IM in the theory of purposeful behavior (Barrick et al., 2013). We will therefore explore the extent to which semantic compliance relates to salary levels:

H2: By job type, semantic compliance of job type holders differ by salary levels.

In establishing the second independent rating, we look for the job characteristics as perceived by others. This is our second independent data point and replicates the original study of Hackman and Oldham (1976), who also used an external panel of raters to test JCM. A fundamental condition for influencing motivation by designing or crafting jobs is that there are some characteristics that will be apparent to most people, whether they hold the actual job or not. In the next hypothesis, we repeat this but look for differences in semantic compliance instead of motivational levels. On the other hand, the general public’s perception of the characteristics and status of a job may in part be influenced by its market value, as indicated by salary levels. Our aim is to show that:

H3: By job type, external panel opinions of job characteristics differ by semantic compliance of job type holders, even when controlled by salary.

Finally, one may ask if these dynamics are of practical importance. If situational characteristics influence both the measurement values *and* the measurement instruments, one must expect that differences in motivational levels between groups may be evened out by the interpretative sense-making process (Barrick et al., 2013). People with different work contexts may make similar ratings of their motivational level. As noted by the authors of JCM and SDT, the general public perceives notable differences in job characteristics across society (Oldham and Hackman, 2010; Deci et al., 2017). We therefore expect a panel of raters to rate the job characteristics as more diverse than the job holders will rate their motivational levels:

H4: The standard deviation in the panel’s job characteristics will show a greater dispersion of scores than the dispersion of self-rated motivational scores.

## Materials and Methods

The following sections describe the source of the data collected, measures used and analyses employed. Each is described in detail.

### Data

The data used in this study represent four completely independent sources. We gathered self-reported levels of motivation from 399 respondents holding 18 different job types. In this context, we want to point out that we use the label “job type” as a simple descriptor of the work situations and characteristics that normally apply to holders of such jobs. Next, we obtained a panel of 30 persons rating the various job characteristics for each of the job types. The public income statistics were yet another dataset. Finally, the fourth dataset was made up of LSA semantic similarity indices computed on the item texts alone.

#### Participants

The original study of Hackman and Oldham (1976) claimed to survey a broad range of job characteristics, but the actual range of these characteristics was not described and seems as if their samples were from varying professions within the companies that participated in the survey. To test our hypotheses, we chose to aim for the broadest possible range of job characteristics within a society. Our self-report motivation sample therefore consisted of 399 persons from 18 job types. We aimed for equal sizes for ease of analysis, but this was difficult as the willingness to participate varied greatly across the job types. The number of 20 respondents in each group was chosen partly to balance the most reluctant groups of participants, and partly because groups of this size have previously been found to display consistent semantic behavior (Arnulf et al., 2018a, d; Arnulf and Larsen, 2020). We offer here a brief description of the job types and how respondents were enlisted:

Chief Executive Officers (CEOs) are very well paid, and wield much power. They responded willingly and our sample contains some of Norway’s most high-profiled CEOs. As a contrast, we obtained a sample of street magazine vendors. These are generally drug addicts or other socially disadvantaged people who are given this job as a respectable means to make a living. They earn very little and only based on their sales. Others who earn little are a sample of volunteers from NGOs who enlist because of their support for a cause. Similarly ideologically inclined but also paid were a group of priests from the Church of Norway. As an assumed contrast to the purely value-based jobs, we enlisted a group of sex workers. This posed some difficulties as buying (but not selling) sex is illegal in Norway, leading to some reluctance in accepting contact. Some of the subjects were working in the streets and surveyed in a sheltering home, while others were contacted through online escort services. Another group was made up of purely professional soldiers, that is, who had been in paid combat service not as a part of mandatory military service or as part of a planned military career. Many of these did not want to give away their e-mail addresses, responding instead to paper and pencil versions of the survey. These groups were not easy to reach, but answered generously once they understood our request. We also contacted professions with high performance pressure such as professional athletes, artists, and stock brokers. The other groups could be seen as less extreme in job characteristics, such as car sales representatives, farmers, lawyers, morticians, dancers, and photographers. Taken together, we assumed that these groups would represent the true variation of motivationally relevant job characteristics in society. The cleaners and street magazine sellers were least willing to participate. The priests and the farmers were most enthusiastic and expressed happiness that someone was interested in their working conditions.

In total, we contacted 1,051 individuals as possible job holders but of these, only 504 potential respondents were identified to be in our target groups and asked to fill out a survey. Our 399 responses make up 79% of these 504 potential respondents. Table 1 shows the 18 job types with the number of participants and gender distribution. Due to the sensitive nature of some professions, we refrained from asking about personal data from the respondents, but we did ask about gender even if this was not mandatory. Several groups appeared inclined to skip the gender question, resulting in large numbers of “unknown.”

**TABLE 1:** Number of participants and distribution of gender for each job type.

Job type	Male	Female	Unknown	Total
Artist	10	12	0	22
Athlete	6	8	6	20
Bouncer	3	0	14	17
Car Sales Rep.	22	1	0	23
CEO	17	7	0	24
Cleaner	4	8	1	13
Dancer	2	10	8	20
Doctor	7	6	9	22
Farmer	8	4	27	39
Lawyer	13	7	0	20
Magazine Seller	13	5	0	18
Mortician	13	8	0	21
Photographer	10	11	0	21
Priest	23	11	4	38
Sex Worker	1	12	9	22
Soldier	10	6	3	19
Stockbroker	16	2	2	20
Volunteer	3	3	14	20
Total	181	121	97	399
% of Total	45.4	30.3	24.3	100

#### Panelists

Following the approach of Hackman and Oldham (1976) the job characteristics were rated by an external evaluation panel. The panel consisted of 30 individuals working in Norway with no relationships to the first sample or knowledge about the purpose of the study. The panel was recruited as a convenience sample from the researchers’ own network. The inclusion criteria aimed simply to attain a representative group of adults with knowledge about the working world with dispersed demographics, resulting in 53% females with an age span of 17–62 years. The sample rated the job types on the JCM dimensions in order to obtain independent evaluations of perceived job characteristics associated with each job type. The panel members individually filled out a Norwegian-language web-based or paper survey.

#### Income

Our source of information about income for the job types was the Norwegian National Statistics Bureau, SSB. These data were not collected from the respondents themselves, but consist entirely of the average income levels as listed by SSB in 2018.

#### Semantic Similarity Indices

The text of all the survey items was projected into a semantic space that we created out of texts from journal articles in the field of psychology. We termed this semantic space “psych” to denote its semantic heritage from psychological texts. This procedure returned a list of semantic cosines for ([50^∗^49]/2) = 1,225 unique item pairs. This is the semantic equivalent of the correlation matrix (Arnulf et al., 2018d), and we will refer to this as LSA cosines or semantic similarity indices. The software for creating semantic spaces and projecting texts can be found as packages in Python (Anandarajan et al., 2019) or R (Günther et al., 2015; Wild, 2015; Gefen et al., 2017).

Semantic values raise a problem with negative correlations, because the cosines almost never take negative values. When they do, the negative sign can be read simply as very distant in the semantic matrices. Negative values do not indicate “opposite” as in correlations, where “like” is the opposite of “not like.” In this study, we handled negative correlations by reverse-scoring all negatively worded items. This is often done with reversed items within scales. Additionally, to avoid the problem of negative cosines, we also reverse-scored two scales that are always negatively related to all the others, Turnover intention (TI) and economic exchange (EE).

### Likert-Scale Measures

We will here describe in detail the self-rating scales on eight motivational constructs, along with the measurement instrument for job characteristics and the data on pay levels. Since motivation is a latent construct, we have chosen to include measures of motivational states together with their purported outcomes. A broader set of items allows a clearer analysis of semantic influences. Also, the inclusion of the outcomes lets us detect if the motivational effects vary along the motivational states as semantically predicted.

#### Self-Rated Motivation

We assembled a series of eight commonly used scales for measuring motivation in conjunction with self determination theory (SDT), totaling 50 items. All items were measured using a five-point Likert response scale ranging from 1 (strongly disagree) to 5 (strongly agree) and administered through a web- and paper-based survey. The first three variables – intrinsic motivation, with social and EE – can be seen as expressions of motivational states. The next four – citizenship behaviors, TI, work effort (WE) and work quality (WQ) – can be seen as outcome measures. The measures in the questionnaire are as follows.

Intrinsic motivation is defined as to “perform an activity for itself, in order to experience the pleasure and satisfaction inherent in the activity” (Kuvaas, 2006b, p. 369). This was assessed with a six-item scale developed by Cameron and Pierce (1994). One example item is ‘My job is so interesting that it is a motivation in itself.’

Social exchange (SE) entails “unspecified obligations such that when an individual does another party a favor, there is an expectation of some future return. When the favor will be returned, and in what form, is often unclear” (Shore et al., 2006, p. 839). In contrast, EE involves transactions between parties that are not long-term or on-going but encompass the financial oriented interactions in a relationship. The constructs SE and EE were measured by a 16-item scale developed and validated by Shore et al. (2006) and previously used in a Norwegian context (Kuvaas and Dysvik, 2009). The SE and EE constructs were each measured with eight items. An example EE item is ‘I do not care what my organization does for me in the long run, only what it does right now.’ An example SE item is ‘The things I do on the job today will benefit my standing in this organization in the long run.’

Organizational citizenship behavior (OCB) is defined as the “individual behavior that is discretionary, not directly or explicitly recognized by the formal reward system, and that in aggregate promotes the effective functioning of the organization” (Organ, 1988, p. 4). The construct was assessed with a seven-item measure validated by Van Dyne and LePine (1998). An example item is ‘I volunteer to do things for my work group.’

Affective organizational commitment (AOC) can be defined as “an affective or emotional attachment to the organization such that the strongly committed individuals identifies with, is involved in, and enjoys membership in, the organization” (Meyer and Allen, 1997, p. 2). AOC was measured with six items previously used by Kuvaas (2006b), originally developed by Allen and Meyer (1990). A sample item is ‘I really feel as if this organization’s problems are my own.’

Turnover intention may be defined as “behavioral intent to leave an organization” (Kuvaas, 2006a, p. 509). The five items were retrieved from Kuvaas (2006a). One example item is ‘I will probably look for a new job in the next year’.

Work quality is defined as “quality of the output” (Dysvik and Kuvaas, 2011, p. 371), while WE is defined as “the amount of energy an individual put into his/her job” (Buch et al., 2012, p. 726). Kuvaas and Dysvik (2009) developed a scale with five items for each. A sample WE item is ‘I often expend extra effort in carrying out my job,’ while a sample WQ item is ‘I rarely complete a task before I know that the quality meets high standards.’

#### Job Characteristics Model (JCM)

Eleven different characteristics connected to JCM were identified and operationalized as single items for each job type, and rated by our panel (see Table 2). The items for autonomy, feedback, skill variety, task identity and task significance were developed by Hackman and Oldham (1975) as part of their original research. As outlined by Barrick et al. (2013), and also as indicated by a later review of JCM (Oldham and Hackman, 2010), there are more characteristics that may activate motivational states than what was originally assumed, particularly related to prestige, power, and other social characteristics. We therefore asked the panel to also rate the jobs on work-life balance, power, safety/danger, prestige, and relatedness (Delecta, 2011; Hauser and Warren, 2012; Ryan and Deci, 2017). To avoid a cumbersome number of items for the panel to fill out, we followed the original procedure from JCM using single-item questions about characteristics for each profession (Hackman and Oldham, 1976).

**TABLE 2:** Job characteristic descriptions and items for the external evaluation panel.

Job Characteristic	Description	Question asked for each job type
Autonomy	“The degree to which the job provides substantial freedom, independence, and discretion to the individual in scheduling the work and in determining the procedures to be used in carrying it out” (Hackman and Oldham, 1975, p. 162).	The job gives a person considerable opportunity for independence and freedom in how he or she does the work.
Feedback	“The degree to which carrying out the work activities required by the job results in the individual obtaining direct and clear information about the effectiveness of his or her performance” (Hackman and Oldham, 1976, p. 258).	To what extent does doing the job itself, managers or co-workers or cooperation with others provide the person with information about his or her work performance?
Pay	Fixed regular payment an employee receives as a compensation for the employment.	Do you think this profession would be a nice profession if money had not been a problem?
Power	“Absolute capacity of an individual agent to influence the behavior or attitudes of one or more designated target persons at a give point in time” (Yukl, 2012b, p. 189).	Do you think this profession implies the ability to execute power?
Prestige	“By educational attainment, by occupational standing, by social class, by income (or poverty), by wealth, by tangible possession” (Hauser and Warren, 2012).	I would have bragged about having this profession to others.
Relatedness	“Both experiencing others as responsive and sensitive and being able to be responsive and sensitive to them – that is, feeling connected and involved with others and having a sense of belonging” (Ryan and Deci, 2017, p. 86).	Do you think this profession contains meaningful relationships with other people?
Safety/danger	Risks of being injured at work.	Do you think this profession is exposed to any risk/danger?
Skill variety	“Degree to which a job requires a variety of different activities in carrying out the work, involving the use of different skills and talents of the employee” (Hackman and Oldham, 1975, p. 161).	How much variety is there in the job? That is, to what extent does the job require a person to do many different things at work, using a variety of his or her skills and talents?
Task identity	“The degree to which the job requires completion of a ‘whole’ and identifiable piece of work; that is, doing a job from beginning to end with a visible outcome” (Hackman and Oldham, 1975, p. 162).	The job provides a person with the chance to finish completely any work he or she starts.
Task significance	“The degree to which the job has a substantial impact on the lives or work of other people, whether in the immediate organization or in the external environment” (Hackman and Oldham, 1975, p. 161).	In general, how significant or important is the job? That is, are the results of the person’s work likely to significantly affect the lives or well-being of other people?
Work-life balance	“An individual’s ability to meet their work and family commitments” (Delecta, 2011, p. 187).	Do you think this profession enables a person to balance work and leisure?

### Analyses

We began our analyses by computing semantic compliance so that we could build our participant database. Semantic compliance (or similarity with the semantic matrix) was created for each participant by regressing the absolute difference between item scores (i.e., individual item distance matrix) on corresponding LSA cosines that were derived from the psych semantic space (i.e., semantic similarity matrix) and saving the *unstandardized slope* (Benichov et al., 2012; cf. Arnulf et al., 2018d).

A series of regression analyses were conducted to determine to what extent job type and the interaction between job type and semantic compliance explained the variance in motivation scores, thereby allowing us to simultaneously look at differences between and within job type as predicted in H1. To interpret the regression effects, we used regression commonality analysis (cf. Nimon et al., 2008). We then aggregated self-reported levels of motivation and external panel opinions of job characteristics by job type, and explored first how salary levels predicted semantic compliance (H2), next how job characteristics as rated by the external panel predicted semantic compliance (H3), and finally if the dispersion of scores was different in the panel and self-rating groups (H4).

## Results

We first present the overall score levels and relationships for the participant data (see Table 3) before proceeding to the hypotheses analyses. Across all job types, semantic compliance had a mean of -0.16 (*SD* = 0.4). This implies that on average, participants showed a tendency to be semantically compliant. Further, semantic compliance was most highly related to score levels on TI, affective commitment (AC), WQ, and IM. Note that TI and EE are reverse-scored. The alpha coefficients of all scales were generally high (0.75 − 0.90) and they generally correlate quite highly with each other. In particular, TI tends to correlate highly with all other scales, while WQ usually displays the lowest correlations with other scales.

**TABLE 3:** Correlation matrix and descriptive statistics for semantic compliance and self-reported levels of motivation.

	SC	AC	EE^*a*^	IM	OCB	SE	TI^*a*^	WE	WQ
AC	0.26	0.75							
EE	0.03	0.52	0.84						
IM	0.14	0.59	0.55	0.90					
OCB	–0.01	0.34	0.26	0.26	0.87				
SE	–0.03	0.47	0.28	0.45	0.35	0.80			
TI	0.38	0.50	0.48	0.62	0.13	0.41	0.89		
WE	0.11	0.37	0.28	0.53	0.39	0.31	0.31	0.78	
WQ	0.15	0.13	0.05	0.29	0.38	0.21	0.16	0.53	0.75
*M*	–0.16	3.73	3.96	4.30	3.95	3.71	4.04	4.26	3.92
*SD*	0.40	0.79	0.83	0.76	0.65	0.71	1.02	0.60	0.54

*^*a*^Reverse coded. Coefficient alpha along the diagonal. SC, semantic compliance. AC, affective commitment. EE, economic exchange. IM, intrinsic motivation. OCB, organizational citizenship behavior. SE, social exchange. TI, turnover intention. WE, work effort. WQ, work quality.*

### Hypothesis 1

Hypothesis 1 considered whether *self-reported levels of motivation differed by job type and the interaction between job type and semantic compliance*. To test H1, we ran regression analyses on each eight motivational scales using job type and the interaction between job type and semantic compliance as predictors. The results can be seen in Table 4. Across most motivational scales, job type and the interaction between job type and semantic compliance contributed significantly to the explained variance, supporting H1. While job type alone mostly has a greater explanatory effect on most score levels than the interaction between job type and semantic compliance, this relationship varies visibly across the scales. In the case of TI, the interaction between job type and semantic compliance predicts motivational level better than job type.

**TABLE 4:** Regression results for motivation measures by job type (JT) and the interaction of job type and semantic compliance (SC).

	AC			EE^a^			IM			OCB			SE			TI^a^			WE			WQ		
Job Type	*b*_*0*_	*b*_*1*_	*p*	*b*_*0*_	*b*_*1*_	*p*	*b*_*0*_	*b*_*1*_	*p*	*b*_*0*_	*b*_*1*_	*p*	*b*_*0*_	*b*_*1*_	*p*	*b*_*0*_	*b*_*1*_	*p*	*b*_*0*_	*b*_*1*_	*p*	*b*_*0*_	*b*_*1*_	*p*
Artist	3.46	1.88	**<0.01**	4.24	0.31	0.55	4.86	0.27	0.59	3.15	1.25	**0.01**	3.40	0.95	0.06	4.50	0.75	0.25	4.62	0.22	0.61	4.31	0.50	0.21
Athlete	3.77	0.21	0.51	3.90	0.08	0.82	4.43	0.12	0.69	3.97	–0.15	0.61	3.62	–0.40	0.21	3.91	1.61	**<0.01**	4.48	0.16	0.57	3.94	–0.01	0.97
Bouncer	3.32	1.31	**<0.01**	3.46	0.99	**<0.01**	3.95	1.46	**<0.01**	4.17	–0.01	0.95	3.41	1.10	**<0.01**	3.43	1.75	**<0.01**	4.25	–0.05	0.81	3.99	–0.25	0.23
Car Sales Rep	3.79	0.41	0.26	3.93	–0.15	0.68	4.36	0.36	0.30	4.38	–0.27	0.41	4.12	–0.39	0.28	4.17	0.77	0.09	4.44	–0.08	0.80	¡0.01	0.66	**0.02**
CEO	4.16	–0.09	0.79	4.32	–0.71	**0.04**	4.71	–0.21	0.52	4.30	–0.22	0.48	4.06	–0.48	0.16	4.66	0.35	0.42	4.45	0.18	0.54	3.90	0.02	0.94
Cleaner	2.76	0.32	0.42	2.97	–0.24	0.55	2.91	–0.45	0.23	3.77	–0.59	0.09	3.30	–0.72	0.06	2.89	0.61	0.22	3.73	–0.09	0.78	3.78	–0.50	0.10
Dancer	3.99	0.39	0.38	4.07	–0.51	0.26	4.47	0.18	0.66	3.81	0.08	0.83	3.73	–0.05	0.91	3.85	1.11	**0.05**	4.33	0.37	0.32	3.84	0.67	**0.05**
Doctor	3.75	0.72	0.05	4.38	0.53	0.15	4.51	0.17	0.62	3.98	0.34	0.29	3.72	–0.21	0.55	4.40	1.19	**0.01**	4.23	0.50	0.10	3.79	0.20	0.48
Farmer	3.99	0.26	0.35	4.32	0.03	0.90	4.40	0.02	0.93	3.96	–0.17	0.51	3.71	–0.50	0.07	4.40	0.13	0.71	4.26	0.22	0.35	3.78	0.11	0.63
Lawyer	3.38	–0.50	0.24	4.12	0.33	0.44	3.93	–0.26	0.52	3.97	–0.39	0.30	3.86	–0.10	0.81	3.92	1.68	**<0.01**	4.26	0.08	0.83	3.91	–0.02	0.95
Magazine Seller	3.20	1.32	**<0.01**	2.92	0.39	0.29	3.63	0.84	**0.01**	3.33	–0.05	0.88	3.27	0.74	**0.04**	3.37	1.59	**<0.01**	3.92	0.26	0.39	3.64	0.01	0.96
Mortician	3.93	0.64	**0.04**	4.26	–0.42	0.19	4.43	0.12	0.68	4.30	0.19	0.50	4.11	0.14	0.66	4.39	0.61	0.13	4.50	0.21	0.43	4.22	0.58	**0.02**
Photographer	4.37	0.60	0.22	4.34	0.21	0.68	4.77	0.10	0.83	3.89	0.88	**0.04**	4.14	–0.65	0.19	4.58	0.18	0.77	4.58	0.40	0.34	4.19	0.58	0.13
Priest	4.06	0.30	0.33	4.41	–0.01	0.98	4.60	0.19	0.52	3.94	0.42	0.13	3.60	–0.35	0.25	4.33	0.70	0.07	4.01	0.50	**0.05**	3.70	0.33	0.17
Sex Worker	3.21	–0.06	0.86	3.12	–0.34	0.31	3.85	–0.19	0.55	3.66	–0.11	0.71	3.49	–0.16	0.63	3.66	–0.29	0.48	3.64	–0.16	0.58	3.97	0.08	0.76
Soldier	3.64	0.43	0.15	4.09	–0.08	0.79	4.26	0.33	0.25	4.33	0.25	0.35	3.54	–0.31	0.29	3.69	1.12	**<0.01**	4.30	0.16	0.53	4.01	0.55	**0.02**
Stockbroker	3.20	0.73	0.08	3.04	0.14	0.74	3.89	0.19	0.63	3.59	0.18	0.62	3.65	0.53	0.19	3.61	1.89	**<0.01**	4.22	–0.06	0.86	3.82	0.09	0.77
Volunteer	3.80	–0.82	0.09	4.26	–0.16	0.75	4.34	–0.59	0.21	4.29	–0.20	0.64	3.73	–0.92	0.06	4.25	–0.67	0.29	4.35	0.14	0.74	3.89	0.49	0.20
*F*(35,363)			6.15			7.21			6.69			3.81			3.13			6.88			2.81			2.56
*R*^2^			0.37			0.41			0.39			0.27			0.23			0.40			0.21			0.20
CC_JT_			0.23			0.35			0.28			0.23			0.13			0.18			0.18			0.10
CC_JT:SC_			0.13			0.04			0.08			0.05			0.10			0.18			0.03			0.07
CC_JT,*JT*:SC_			0.02			0.02			0.03			–0.01			0.01			0.04			0.01			0.03

*^*a*^Reverse coded. AC, affective commitment. EE, economic exchange. IM, intrinsic motivation. OCB, organizational citizenship behaviors. SE, social exchange. TI, turnover intention. WE, work effort. WQ, work quality. *P* ≤ 0.05 shown in bold. CC, commonality coefficient. In some cases, ∑CC≠*R*^2^due to rounding errors.*

**FIGURE 1:**
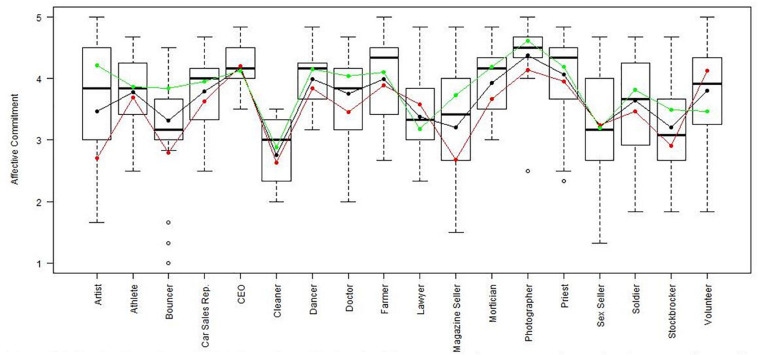
Affective commitment by job type. Green, black, and red lines respectively represent estimates based on semantic compliance of 0.24 (*M* + 1 *SD*), –0.16 (*M*), and –56 (M – 1 *SD*).

**FIGURE 2:**
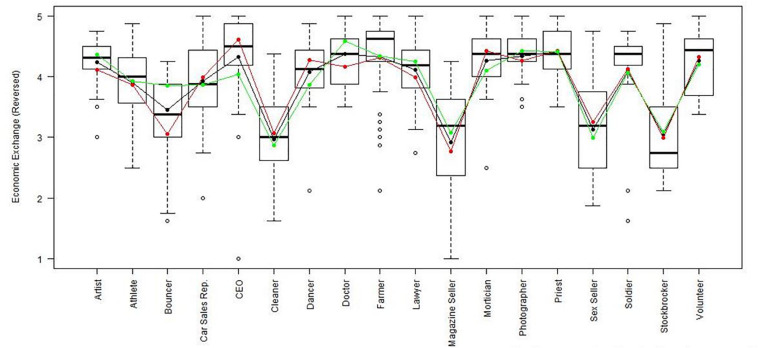
Economic exchange (Reversed) by job type. Green, black, and red lines respectively represent estimates based on semantic compliance of 0.24 (*M* + 1 *SD*), –0.16 *(M)*, and –0.56 (*M –* 1 *SD*).

**FIGURE 3:**
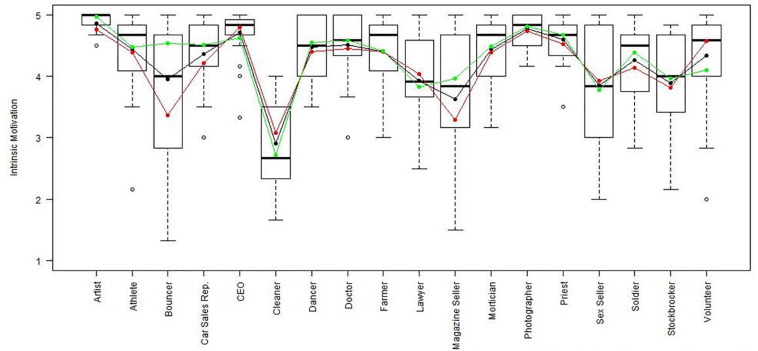
Intrinsic motivation by job type. Green, black, and red lines respectively represent estimates based on semantic compliance of 0.24 (*M* + 1 *SD*), –0.16 (*M*), and –0.56 (*M –* 1 *SD*).

**FIGURE 4:**
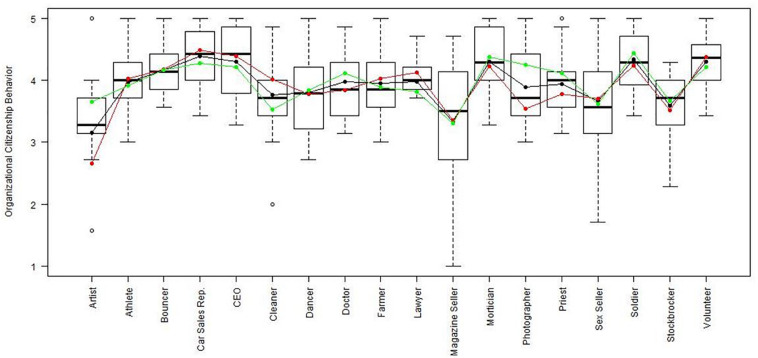
Organizational citizenship behavior by job type. Green, black, and red lines respectively represent estimates based on semantic compliance of 0.24 (*M* + 1 *SD*), –0.16 (*M*), and –0.56 (*M –* 1 *SD*).

**FIGURE 5:**
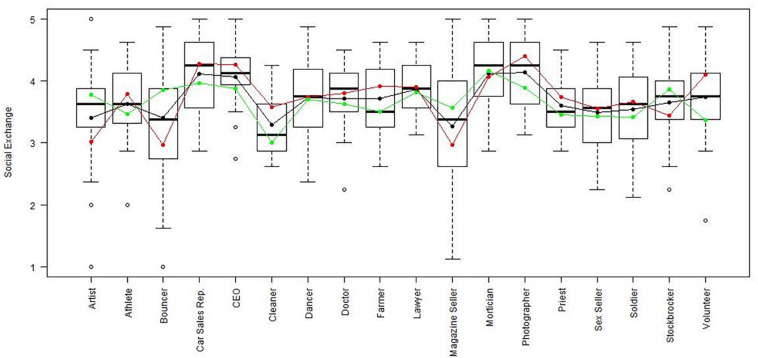
Social exchange by job type. Green, black, and red lines respectively represent estimates based on semantic compliance of 0.24 (*M* + 1 *SD)*,−0.16 (*M*), and –0.56 (*M–1 SD*).

**FIGURE 6:**
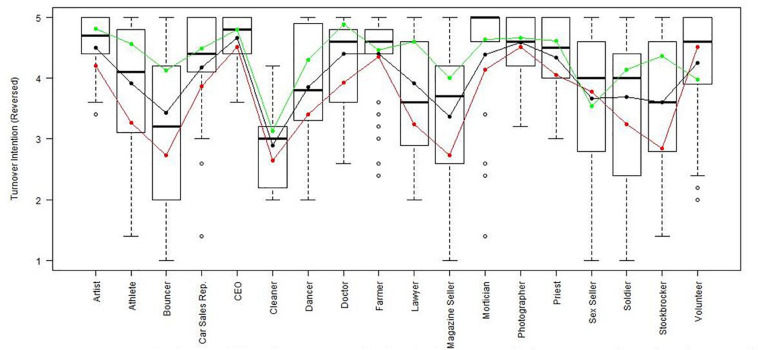
Turnover intention (Reversed) by job type. Green, black, and red lines respectively represent estimates based on semantic compliance of 0.24 (*M* + 1 *SD*), –0.16 (M), and –0.56 (*M –* 1 *SD*).

**FIGURE 7:**
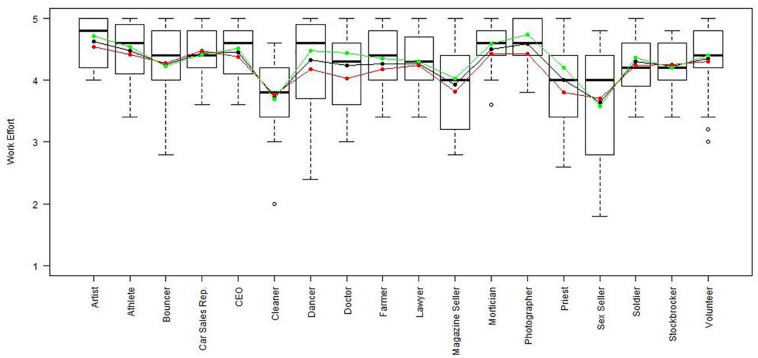
Work effort by job type. Green, black, and red lines respectively represent estimates based on semantic compliance of 0.24 (*M* + 1 *SD*), –0.16 (*M*), and –0.56 (*M* – 1 *SD*).

**FIGURE 8:**
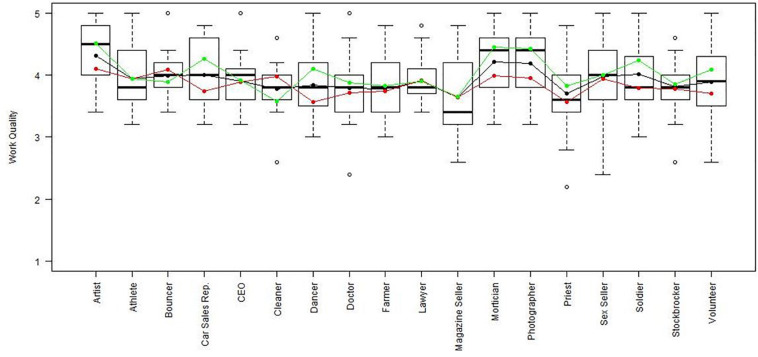
Work quality by job type. Green, black, and red lines respectively represent estimates based on semantic compliance of 0.24 (*M* + 1 *SD)*,−0.16 (*M*), and –0.56 (*M –* 1 *SD*).

Using the regression results, we also looked at whether respondents with high, average or low semantic compliance had significantly different score levels on each scale (see Figures 1–8). It appears that some groups display more semantic disparities than others, and some scales also create greater differences within job types than others. Interestingly, each profession differentiated in the association between their semantic compliance and self-reported levels of motivation for at least one measure.

The largest differentiation in semantic compliance takes place in responding to TI. Eight job types display significant differences in score levels based on their semantic compliance: athletes, bouncers, dancers, doctors, lawyers, magazine sellers, soldiers and stockbrokers. Next, for AC, there are five groups displaying significant differences in score level depending on semantics: artists, bouncers, doctors, magazine sellers, and morticians.

Conversely, some scales do not seem to elicit much within-group differences. For WE, only priests seem to differentiate. For EE, only bouncers and CEOs differentiate, and for IM, only bouncers and magazine sellers do.

The box plot for turnover intention also shows a general trend for the whole sample, namely, that higher semantic compliance is often related to somewhat lower or at least moderated mean score levels (note that turnover intention as a scale is reverse-scored in our analysis). There are only two notable differences, volunteers and sex workers, whose values are not significantly different from zero.

Two interesting cases are WQ and WE. These are the scales where the differences between groups are least pronounced. There are still discernible within-group differences in score levels and semantic compliance, enough to make high scorers less semantically compliant. In the case of WQ, where all groups score about the same, semantics explain almost as much unique variance as the score level differences (35% vs. 49% of the explained variance).

Together, Table 4 and the box plots in Figures 1–8 show that different job types will have different impacts on the relationship between semantics and score levels. There is no single, simple relationship between the two. Instead, the same groups of items seem to be interpreted so differently within and between groups that there will be significant differences in score levels depending on these differences. Looking at the relationship between semantics and motivational scales, a pattern emerges that may be due to semantic uncertainty where respondents differ.

Even if the interactions are complex, there are also some more linear relationships between semantics and motivational levels. Table 5 sorts mean self-reported levels of motivation from least to most semantically compliant. Aggregated by job type, the mean motivational measures of turnover intention and OCB were the most semantically related but in opposite directions and the mean motivational measures of economic exchange and WE were the least semantically related (see Table 6). Taken together, these findings support H1.

**TABLE 5:** Job type self-reported levels of motivation sorted by similarity compliance (SC).

		Motivational Measures							
Job type	SC	AC	EE^a^	IM	OCB	SE	TI^a^	WE	WQ
*Artist*	−0.04	3.68	4.27	4.89	3.30	3.51	4.59	4.65	4.36
*Mortician*	−0.06	3.99	4.22	4.44	4.32	4.13	4.45	4.52	4.28
*Mag. Seller*	−0.07	3.32	2.96	3.70	3.33	3.33	3.51	3.94	3.64
*Farmer*	−0.07	4.01	4.33	4.41	3.94	3.67	4.42	4.28	3.79
*Car Sales Rep.*	−0.08	3.83	3.91	4.39	4.36	4.09	4.23	4.43	4.05
*Cleaner*	−0.08	2.78	2.95	2.87	3.73	3.24	2.94	3.72	3.74
Photographer	−0.11	4.40	4.35	4.78	3.94	4.11	4.59	4.60	4.22
Priest	−0.12	4.07	4.41	4.61	3.96	3.59	4.36	4.02	3.71
Volunteer	−0.15	3.79	4.26	4.33	4.29	3.73	4.24	4.35	3.90
Dancer	−0.15	3.99	4.07	4.48	3.81	3.73	3.86	4.33	3.84
Sex Worker	−0.16	3.21	3.13	3.85	3.66	3.49	3.66	3.64	3.97
Stockbroker	−0.16	3.20	3.04	3.89	3.59	3.65	3.60	4.22	3.82
CEO	−0.18	4.17	4.33	4.72	4.30	4.07	4.65	4.44	3.90
**Athlete**	−0.27	3.75	3.89	4.42	3.99	3.67	3.73	4.46	3.94
**Doctor**	−0.31	3.64	4.30	4.48	3.93	3.75	4.22	4.15	3.76
**Soldier**	−0.31	3.57	4.11	4.21	4.29	3.59	3.52	4.27	3.93
**Lawyer**	−0.32	3.46	4.06	3.98	4.04	3.88	3.64	4.25	3.91
**Bouncer**	−0.33	3.10	3.29	3.70	4.17	3.22	3.13	4.26	4.04
*SD*		0.42	0.54	0.49	0.33	0.28	0.52	0.28	0.20

*^*a*^Reverse coded. AC, affective commitment. EE, economic exchange. IM, intrinsic motivation. OCB, organizational citizenship behaviors. SE, social exchange. TI, turnover intention. WE, work effort. WQ, work quality. SC, semantic compliance. Top six least semantic *italicized and underlined*. Top five most semantic bolded.*

**TABLE 6:** Correlations between self-reported levels of motivation, semantic compliance, salary, and panel responses of job characteristics aggregated by job type.

	Motivational measures											
Measures	AC	EE^a^	IM	OCB	SE	TI^a^	WE	WQ	SC	ρ_*SC*_	ρ_*Salary*_	ρ_*SC.Salary*_
**Job characteristic**												
Autonomy^b^	0.61	0.53	0.65	–0.24	0.30	0.70	0.45	0.21	0.33	0.30	0.04	0.42
Feedback^b^	0.46	0.56	0.56	0.38	0.66	0.36	0.59	0.21	–0.40	–0.48	0.49	–0.25
Work without pay	0.59	0.70	0.65	0.13	0.46	0.58	0.63	0.16	–0.13	–0.09	0.23	0.08
Power	0.21	0.45	0.36	0.34	0.31	0.23	0.32	–0.01	–0.64	–0.64	0.65	–0.38
Prestige	0.43	0.56	0.51	0.19	0.42	0.37	0.53	–0.06	–0.38	–0.40	0.50	–0.12
Relatedness	0.50	0.65	0.48	0.31	0.41	0.49	0.18	–0.08	–0.15	–0.03	0.33	0.24
Safety/danger	–0.25	–0.22	–0.15	0.01	–0.34	–0.36	–0.35	–0.21	–0.56	–0.62	0.46	–0.48
Skill variety^b^	0.46	0.64	0.57	0.20	0.40	0.50	0.29	0.01	–0.35	–0.34	0.71	0.21
Task identity^b^	0.18	0.04	0.10	–0.22	0.30	0.26	0.22	0.43	0.42	0.38	–0.21	0.33
Task significance^b^	0.28	0.61	0.32	0.38	0.27	0.35	0.16	–0.12	–0.36	–0.24	0.47	0.08
Worklife balance	0.02	0.04	–0.02	0.03	–0.13	0.11	0.12	0.32	0.43	0.44	–0.70	< 0.01
SC	0.23	–0.02	0.11	–0.34	0.12	0.40	0.04	0.18	1.00	1.00	–0.63	
ρ_*SC*_	0.31	0.11	0.24	–0.25	0.05	0.42	0.25	0.06				
ρ_*Salary*_	0.13	0.32	0.12	0.24	0.22	0.10	–0.21	–0.17				
ρ_*SC.Salary*_	0.52	0.43	0.41	–0.13	0.25	0.62	0.16	–0.06				

*Unless otherwise noted, correlations are Pearson’s *r*. ^*a*^Reverse coded. AC, affective commitment. EE, economic exchange. IM, intrinsic motivation. OCB, organizational citizenship behavior. SE, social exchange. TI, turnover intention. WE, work effort. WQ, work quality. SC, semantic compliance. ^*b*^Characteristics associated with Hackman and Oldham (1975) job characteristic model. Given a sample size of 18, absolute correlation coefficients of 0.71 are statistically significant at alpha = 0.001; 0.59 at alpha = 0.01, 0.47 at alpha = 0.05, and 0.40 at alpha = 0.10.*

### Hypothesis 2 and 3

Hypothesis 2 and 3 examined data aggregated by job type and considered whether *salary levels* (H2) and *external panel opinions of job characteristics controlled by salary levels* (H3) *differed by semantic compliance of job type holders.* Interestingly, there are significant relationships between the four independent sources – national salary levels, panel-rated characteristics, self-rated motivation and semantic values. Group means for semantic compliance, salary, and the panel-rated characteristics are listed in Table 7, together with the inter-rater reliabilities of the panel characteristics ratings. The ICCs of the panel ratings are all above 0.92 except for the variable *task identity*, which is only 0.52. Salary turns out to be significantly related to semantic compliance of the job holders, as the rank-order correlation between semantic compliance and *salary* is −0.63. This supports H2. Table 7 also shows a tendency for groups of high and low scores to cluster along the continuum made up by semantic compliance and income.

**TABLE 7:** Job type panel responses of job characteristics and salary sorted by similarity compliance (SC).

		Job Characteristics											
Job Type	*SC*	AU	FB	WPay	PWR	PR	RL	RISK	SV	TI	TS	WLB	Salary
*Artist*	*−0.04*	**4.53**	3.47	**3.67**	2.67	3.13	2.93	*1.73*	3.43	**4.03**	3.07	**4.17**	*7,416*
*Mortician*	*−0.06*	*2.37*	3.73	*2.17*	*2.13*	*2.23*	**4.30**	*1.63*	*2.27*	**4.23**	**4.00**	3.70	*40,200*
*Magazine Seller*	*−0.07*	3.20	*2.33*	*1.83*	*1.20*	*2.00*	3.33	**3.10**	*2.03*	*3.60*	*2.43*	3.53	*1,000*
*Farmer*	*−0.07*	**4.10**	*2.60*	3.07	2.20	3.13	*2.60*	2.83	3.57	4.00	3.37	2.60	46,173
*Car Sales Rep.*	*−0.08*	*2.47*	3.67	*2.00*	*1.83*	*1.90*	*2.40*	*1.77*	*2.00*	3.77	*2.10*	**4.23**	*36,275*
*Cleaner*	*−0.08*	*1.97*	*2.57*	*1.47*	*1.27*	*1.73*	*2.17*	*1.57*	*1.50*	**4.07**	*2.73*	**4.03**	*32,370*
Photographer	−0.11	**4.23**	3.70	**3.97**	2.57	3.40	3.23	*1.97*	3.57	**4.20**	2.80	3.83	41,340
Priest	−0.12	3.17	3.37	2.30	3.13	2.27	**4.63**	*1.77*	3.13	*3.67*	**4.27**	3.73	49,800
Volunteer	−0.15	**3.83**	*3.33*	**4.57**	*2.13*	**4.07**	**4.40**	2.07	**3.87**	*3.47*	**4.43**	**4.40**	44,310
Dancer	−0.15	3.47	3.87	3.43	*1.50*	3.57	3.17	2.73	3.10	3.77	*2.40*	2.90	41,500
Sex Worker	−0.16	2.90	*2.50*	*1.23*	*1.33*	*1.07*	*2.57*	**4.50**	3.03	3.90	*2.27*	*2.43*	**77,053**
Stockbroker	−0.16	2.83	3.87	2.50	**3.30**	3.60	*2.13*	2.07	3.03	3.87	2.80	*1.90*	59,165
CEO	−0.18	**4.20**	**4.17**	**3.60**	**4.77**	**4.43**	3.83	2.63	**4.53**	3.80	3.73	*2.17*	**397,232**
**Athlete**	**−0.27**	2.90	**4.40**	3.47	2.77	**4.30**	3.13	2.93	*2.47*	**4.13**	3.13	*2.30*	42,580
**Doctor**	**−0.31**	*2.80*	**4.17**	**3.90**	**4.40**	**4.50**	**4.60**	**3.23**	**4.13**	*3.73*	**4.90**	2.97	**74,450**
**Soldier**	**−0.31**	*2.27*	**3.93**	2.20	**3.70**	3.27	3.33	**4.57**	**4.10**	*3.27*	3.77	*2.27*	**83,000**
**Lawyer**	**−0.32**	3.20	**4.17**	3.50	**4.33**	**3.87**	**4.03**	2.20	**4.10**	3.87	**4.43**	*2.23*	**61,486**
**Bouncer**	**−0.33**	*2.20*	*2.57*	*1.73*	2.97	*1.73*	*2.13*	**3.50**	*2.00*	*3.60*	*2.57*	**4.23**	*37,170*
ICC		0.93	0.92	0.95	0.97	0.96	0.95	0.96	0.94	0.52	0.94	0.96	
*SD*		0.77	0.67	0.99	1.10	1.06	0.86	0.92	0.89	0.26	0.86	0.86	

*AU, autonomy. FB, feedback. WPay, work without pay. PR, prestige. PWR, power. RISK, safety/danger. RL, relatedness. SV, skill variety. TI, task identity. TS, task significance. WLB, work life balance. Top six least semantic *italicized and underlined*. Top five scale values bolded.*

Table 6 shows how the panel’s ratings of job characteristics show strong and significant correlations between job characteristics and motivational levels. In particular, the variables *autonomy*, *feedback*, and *skill variety* were strongly related to motivational variables in the direction suggested by JCM and SDT. Concomitantly, the variable “economic exchange” also correlates highly with the same variables.

Testing H3 raises an issue about sample size. The numbers are based on two samples – one with a panel of 30, the other with 399 respondents – but aggregated by job types the sample size is reduced to 18. The most conservative approach would be to look at relationships with a *p*-level above 0.05, *n* = 18. We find strong correlations between salary levels and the panel’s perception of *power, prestige*, *feedback, worklife balance, safety/danger, skill variety*, and *task significance* (| ρ| ≥ 0.47, *p* ≤ 0.05, Table 6, rightmost columns). Only power and safety/danger as panel rated characteristics appear significantly related to semantic compliance. Controlling for salary, the only significant correlation between job characteristics and semantic compliance is safety/danger. However, considering that the numbers stem from bigger samples, there are sizeable correlations with practical significance. Characteristics originally theorized to predict motivational levels, such as autonomy, feedback, power, relatedness, skill variety and task identity show medium to strong correlations with semantic compliance even after controlling for salary. The lowermost rows in Table 6 show how semantic compliance correlates with the motivational scales themselves (from which the semantic compliance numbers are derived). These numbers are actually significantly lower than the correlations with the panel data (*p* = 0.02, Mann–Whitney test). H3 is therefore at least partly supported.

### Hypothesis 4

The range of average scores on the motivational scales in Table 5 is remarkably narrow. As argued in STSR, a score on a Likert item is en endorsement of a statement, in our case a motivational self-description. If we round the average scores to the nearest integer and replace the integer with the corresponding statement on a motivational scale, the job types would literally describe their motivation in almost the same terms. The differences across job types within each scale exceeds 1 point in only two cases (IM and TI), where the differences do not exceed 2 points. H4 stated that *the standard deviation in the panel’s job characteristics will show a greater dispersion of scores than the dispersion of self-rated motivational scores*. To test this we computed the standard deviation in the panel’s rating of each characteristics across the job types. We then compare this to its counterpart in the self-rated group, by computing the standard deviation of mean scores across motivational levels and job types. The two sets of numbers are displayed at the bottom of Tables 5, 7. It turns out that the variation in the panel’s rating of job characteristics (0.83) is much higher than the variation in self rated motivational levels (0.38, *p* = 0.001 in a Mann–Whitney test), supporting H4.

## Discussion

The purpose of this study was to explore how different professional contexts influence the semantic patterns of responses to motivational items with ensuing consequences for score levels. Our findings supported the predictions from job design theory that levels of motivation differ significantly between job types according to their characteristics (Hackman and Oldham, 1975, 1976), but interestingly, the semantic characteristics of respondents also explained a substantial proportion of the differences in score levels. For most motivation measures, the interaction between job type and semantic compliance explained a substantive amount of unique variance in score levels, supporting H1. This suggests that scholars and scholar-practitioners may be mis-estimating the effect of job type on motivation when using traditional methods that do not consider participants’ tendency to respond semantically.

Our findings imply that respondents from different job types differ substantially in how they perceive and interpret the items. Different job types do not only give people different subjective levels of motivation, but these job types also influence and probably change the meaning of each item. The effect is not a general methodological effect with equal impact across conditions, because some situations seem to alter the meaning of some scales more than others. This demonstrates that the relationship between job characteristics and self-rated motivation is not a two-way relationship. Instead, it is a three-way relationship, depending also on the subjects’ semantic parsing of the items, which will vary systematically both between and within job types. Our finding is in line with the theory of purposeful behavior, which states that job holders will engage in sense-making activities to proactively create meaning in their situations (Barrick et al., 2013).

Since semantics and score levels are practically intertwined and difficult to separate (Arnulf et al., 2018d), the relationship between the two could possibly be interpreted as a methodological artifact such as common method variance (Podsakoff et al., 2012) or endogeneity (Antonakis et al., 2010). For that reason, we introduced two more independent data sources, an external panel and national statistics on salary levels. Interestingly, there was a strong correlation between the salary levels of the job types and the tendency of the job holders to respond semantically compliant.

This probably has several implications. One obvious reason for this finding is that the language in the survey items is most appropriate for people with high income. Another related reason is that high income is correlated with high social status and education, along with the linguistic habits and competence that come from such demographic variables. Among the most semantically predictable groups are highly trained academics such as lawyers and doctors, and athletes who tend to be competitively oriented and intellectually acute (Cooper, 1969). On the other side of the scale, the cleaners in our study had mostly either little education, or many of them were foreigners with high likelihood of lower language skills. One notable exception in the sample was the bouncers, who are not high earners but who scored very high on semantic compliance. This is a group of people who may be trained in using their verbal skills to deal with people. Also, many holders of these jobs in Norway are people who combine this job with taking a higher education, because it often takes place outside of office hours.

Concerning the second external dataset, the panel data, we hypothesized as H3 that this dataset also would be significantly related to semantic compliance – even after controlling for salary level. We found support for this as well, but not as strongly as with the salary level. Generally, semantic compliance was visibly correlated with most of the job characteristics that also influence levels of motivation such as autonomy, feedback, power, prestige, skill variety and task significance. It is also possible to see from the distribution in Table 6 that semantic compliance does seem related to high and low clusters along work characteristics. These effects were generally changed a bit when controlling for the salary levels, but still had visible influence on the groups’ semantic compliance. Moreover, the semantic compliance of the respondents correlated significantly stronger with the panel’s ratings of their jobs than with their own motivational measures. We believe this speaks strongly in favor of the semantic compliance not being a methodological artifact, even if the aggregation on group level only *n* = 18 job types raised issues of statistical significance.

Taken together, our results indicate that job characteristics and salary levels do influence self-rated levels of motivation as found in previous research, but they also influence semantic compliance independently of the score levels. The emerging differences in semantic compliance are interacting with motivational variables and job types and indicate that extensive differences in interpretation of items take place when respondents enter their scores. Job characteristics still pose the most powerful direct influence on differences in motivational levels, but the influence of semantics is sizeable and sometimes even stronger than the job types.

The theoretical and practical relevance of our findings can be seen by comparing the score levels of some of the professional groups. According to their reported score levels, CEOs are just as intrinsically motivated as priests, and claim just as little interest in their pay level. If this were true in an absolute sense, it would obviate any discussion about executive compensation, which probably is an unlikely interpretation (Ellig, 2014; Shin, 2016). Priests and sex workers differ only on 3 out of 8 measures (affective commitment, economic exchange, and IM), despite their possible differences in work values. Stockbrokers and sex workers have no score level differences but have widely different scores on job characteristics such as autonomy, relatedness, skill variety, and task identity. They work with high effort and quality, and all but bouncers, cleaners and photographers rarely think of quitting their jobs. All respondents claim to be more intrinsically motivated than interested in money (with the possible exception of cleaners).

These similarities in score levels or lack of distinct differences pose the question: Are the numerical levels really indicative of the same level of motivation? Do the measures imply invariant quantifications (Mari et al., 2017; Maul et al., 2019), or do the numbers in the responses represent endorsed statements (Drasgow et al., 2015)? Because in the latter case, responses must be treated as context-dependent interpretations.

This question opens the discussion about the nature of semantics in survey research. Words do not have fixed meanings, independent of context (Kay, 1996; Lucy, 1996; Kintsch, 2001; Sidnell and Enfield, 2012). The context of an utterance determines how it is to be understood. As outlined in the quote by Deci et al. in the introduction (Deci et al., 2017, p. 20), people with demanding and demeaning jobs who struggle to support a family and long for days away from work may interpret some items very differently from people who never worry about paying their rents. Items related to IM is probably not indifferent to this context. The reader is invited to imagine a dinner table conversation where someone says: “I work as a priest. I easily get absorbed in my work and do not think much about my income.” Try to change “priest” with any other profession on the list, and most people will get a feeling that the words somehow take on different meanings.

Previous studies have shown the general semantic predictability between the motivational variables involved in this study (Arnulf et al., 2014, 2018a). A general semantic predictability among variables imply that their relationships are given *a priori* with little room to vary (Semin, 1989; Smedslund, 2002; Arnulf, 2020), such that statements about WE and quality are implicated by other statements about motivation. The obverse side of this is that once a subject chooses a value at an entry point on the scale, the values on the other scales will be given or at least restricted in variance (Feldman and Lynch, 1988; Arnulf et al., 2018b). It is striking how most respondents rate their effort and quality in the high ranges. High self-ratings of effort may be everything from true assessments via self-serving biases (Duval and Silvia, 2002), social desirability (Furnham, 1986) and unskilled unawareness (Kruger and Dunning, 1999; Ehrlinger et al., 2008; Sheldon et al., 2014). From a semantic point of view, people who agree on the scores of one variable are also expected to agree on other variables, which is what we find. In this interpretive process, the semantic influences interact with job characteristics to shape the observed scores.

There is a methodological limitation to this process, best observed in the scores of the CEOs. These people with their high incomes are a seeming exception to the rule that higher income creates higher semantic compliance, but this is probably a ceiling effect. Respondents who score very high (or very low) on all items may reduce their semantic predictability due to the restriction of statistical range. In our sample, this may be the case for photographers, CEOs, and priests. Most of these respondents tend to give such consistently high scores that differences between items are obliterated and thereby also most semantic prediction. Where all items are given similar scores, it becomes hard to detect whether the respondent read any differences into them due to restriction of range.

The most semantically predictable participants in each professional group will therefore, with very few exceptions, be the ones who score slightly lower than the others. It is only possible to be semantically predictable for respondents who vary their scores, which by necessity implies the need for some scores to be lower than others, lowering the average score levels.

Lack of semantic predictability can therefore appear due to the following three causes, with different possible remedies. First, the restriction of range in a ceiling effect where respondents are indiscriminately enthusiastic (or disgruntled), along with any other general response set that flattens the interpretation of items. The second possibility would be a lack of verbal acuity – the respondent does not process the items properly, due to a lack of language skills or simply sloppy reading (cf. Arnulf and Larsen, 2019). In this case, the responses would contain noise. A third possibility would be systematic differences in the way items are processed (cf. Arnulf et al., 2018c), which is what we are really looking for here. Our data show signs of all three explanations.

Ceiling- or flooring effects could be avoided by better procedures in selecting items and scale options, for example by using item response theory (IRT) (van Schuur, 2017). Lack of verbal acuity could possibly be avoided by instructing respondents differently. An unpublished master thesis found that semantic compliance tended to increase when respondents were forced to delay responses with a number of seconds after having been exposed to them (Noack and Bonde, 2018). But maybe the most promising way to proceed with this line of research is to systematically assess the differences in semantic compliance the way we have begun here. Our results indicate that differences in semantic compliance is a systematic characteristic in groups, and that the impact of this is possible to assess.

Elaborating on this point, two limitations of our design are important to bear in mind. First, we are only using one single semantic space. This space seems to favor the language usage of high-status, high-income participants. The semantic algorithms here present some sort of a *standard* language usage, against which all other groups are measured. Conceivably, other groups might be predictable using other types of semantic similarity indices or from other semantic spaces. This question is treated in length by Kintsch (2001), who showed that LSA will need special procedures to pick up the usual differences in language parsing that appear in normal human speakers when contexts change. The systematic tendency for the one semantic space that we use here to predict some groups better than others is probably due to systematic differences in how contexts influence the understanding of items.

Secondly, the different professions also differ in which type of motivational scale is most likely to expose their semantic differences. The two artistic professions, artists and photographers, are usually single person businesses in our sample. Being individuals rather than organizations, the two scales commitment (AC) and organizational citizenship (OCB) create big intra-group variance because the meanings of these items may be very different or even contrived for some of them (see Schwarz, 1999). In the same vein, turnover intention (TI) may be difficult to interpret with professions such as athletes and volunteers where the subjects are probably very conscious of the fact that they are not on a lifelong career track. At the extreme end, our magazine sellers and cleaners are mostly people who probably had no initial intention to do this for a living. This could make turnover intention a complex matter for them.

Taken together, this means that semantic predictability is a group characteristic, but one that will matter more on some variables than on others. If we could establish a common ground for determining the semantic patterns of sub-groups, we could also describe the systematic differences in meaning that different groups attribute to different items.

Even if we cannot test these patterns directly for now, we are able to conclude that different groups see the items in different ways and therefore use the items differently to express their perceived motivation. When the items of a scale (or items between scales) combine to form average score levels, the classic psychometric way of treating the data is to view the numbers as indicating a composite variable. If semantics had not played a role, only scale levels would matter. In that case, the score levels could have been taken as indicators of a *dominance* model in attitude strength (Drasgow et al., 2015), because respondents would only differ along motivational levels. Semantic analyses of the items take this a step further and point to how the items are related to each other in terms of meaning. What we see in the patterns of LSA cosines is how likely one response is, given its relationship to the meaning of other responses. In our data, high-status job holders seem to share this view of the items and respond consistently. This consistent choice of responses is what Coombs called “unfolding” (Coombs and Kao, 1960), and which has been experimentally demonstrated to be highly consistent in individuals (Michell, 1994). However, when other groups of respondents display similar average score levels but deviate from the semantically expected, it means that they are sorting the response options differently. In other words, they are making different combinations of response options from the semantically expected.

This goes to the core of Likert’s (1932) original problem – the relationship between stated points of view and their numerical representations. We offer respondents verbal response options (“is it very likely or very unlikely that you will look for a new job?”) that we translate into numbers (1 – 5) and calculate in statistics. After arriving at the numbers, we need to interpret these into words again (“people who are mostly motivated by money are more likely to look for new jobs”). As claimed by Kjell et al. (2019), semantic algorithms may principally allow us to bypass the numbers and stay with the response texts. Looking at Table 5, we rounded up the mean scores to integers to represent statements about motivation. This created a picture where many job types seemed to express their motivation through fairly identical statements. This rounding up of mean scores did not only conceal significant decimal differences between the groups, it also concealed important semantic differences between the professions. The mean level of scales does not show how the mutual ranking of each item may differ between the professions – they may have ranked items differently to create different stories about their work motivation. Moreover, even similar wordings may have different meanings in different contexts. The same score on the same item seems sometimes to have a different meaning if the context differs.

### Limitations

Our present design required that we varied the job types to ascertain reliable variation in the situational factors, but we restricted the variation in the survey scales that we used. All eight scales were somehow related to measuring motivation. The Cronbach’s alpha of all 50 items combined is actually 0.91. With this homogeneous sample of items, the range of semantic differences is also limited. This means that the LSA cosines probably are an under-estimation of the true semantic structure of the survey. The algorithms are, at the current time, still inferior to humans in language parsing, and so the cosines will contain noise and probably miss semantic differences that are important to the human respondents. A semantically diverse survey structure would possibly make the semantic algorithms more sensitive to differences in semantics between groups. Another limitation is the sample size and the lack of cultural variation in the groups. Larger samples and samples spanning more countries than Norway might very well change the observed statistics.

## Conclusion

We set out to examine whether the semantic response characteristics of individuals would vary across groups, and this seems to be the case. Whereas we usually would look at how different work situations or professional characteristics influence motivation, we also find that the same characteristics influence semantic parsing of item texts. Different situations produce different patterns of relating to the texts in a quantifiable way, about half as predictive of motivational levels as the job situations themselves. One may object that the motivational levels are measurements that we intend to produce – levels of motivation. The semantic patterns are not intended outcomes of the surveys and more difficult to interpret. And yet, as we have shown, the motivational levels have shortcomings seen as measurements of motivation. It is not obvious that the same numerical levels of motivation indicate the same subjective situation in different respondents. As Solomon Asch warned in his book *Social Psychology*, “most social acts have to be understood in their setting, and lose meaning if isolated. No error in thinking about social facts is more serious than the failure to see their place and function” (Asch, 1987, p. 61, orig. 1952). This also seems to apply to Likert-scale statements. The context determines the meaning of the items and influences the interpretation of score levels. Our conclusion is therefore that the semantic characteristics of individuals, the way they interpret items and take context into consideration, is a necessary and integral part of survey data.

## Data Availability Statement

The datasets generated for this study are available on request to the corresponding author.

## Ethics Statement

The studies involving human participants were reviewed and approved by the NSD Norsk Samfunnsvitenskapelig Datatjeneste. Written informed consent for participation was not required for this study in accordance with the national legislation and the institutional requirements.

## Author Contributions

JA designed the study, supervised the data collection, and co-wrote the text. KN analyzed the data, producing the tables and figures, and co-wrote the text. KL performed the semantic algorithms and co-wrote the text. CH and MA established the measures, obtained the samples, and made a preliminary analysis of the data.

## Conflict of Interest

The authors declare that the research was conducted in the absence of any commercial or financial relationships that could be construed as a potential conflict of interest.
